# Cardiac Surgery-Associated Acute Kidney Injury: Procedure-Specific Incidence and Impact on Mortality—An Algorithm-Based Big Data Fusion Analysis

**DOI:** 10.3390/medsci14020209

**Published:** 2026-04-23

**Authors:** Nora Goebel, Micha Christ, Nico Schmid, Carmen Heidrich, Joerg Latus, Mark Dominik Alscher, Bartosz Rylski, Moritz Schanz

**Affiliations:** 1 Department of Cardiovascular Surgery, Robert Bosch Hospital, 70376 Stuttgart, Germany; 2 Center for Medical Data Integration, Bosch Health Campus, 70376 Stuttgart, Germany; 3 Department of General Internal Medicine and Nephrology, Robert Bosch Hospital, 70376 Stuttgart, Germany; 4 Robert Bosch Hospital, and Robert Bosch Society for Medical Research, 70376 Stuttgart, Germany

**Keywords:** algorithm-based analysis, cardiac surgery-associated acute kidney injury, data fusion, intensive care, mortality

## Abstract

Background: Acute kidney injury following cardiac surgery is frequent and is associated with increased risks of morbidity and mortality. However, the incidence, severity, and impact of acute kidney injury after cardiac surgery are still underestimated. Therefore, we analyzed our large electronic database for the procedure-specific incidence, characterization, and outcomes of cardiac surgery-associated acute kidney injury (CSA-AKI). Methods: A total of 8564 patients who underwent cardiac surgery at our center between 2017 and 2024 were included. We used an algorithm-based data fusion technique to aggregate data from different electronic health record sources. Data were analyzed regarding acute kidney injury according to the full kidney disease: Improving Global Outcomes (KDIGO) criteria. Results: The patients’ median age was 68 [60; 75] years, and 75.2% were male. The overall incidence of CSA-AKI was 70.5% during the hospital stay, and its development was significantly associated with increased in-hospital mortality rates (5.9% vs. 0.9%, *p* < 0.001). In regression analysis, all AKI stages were independent predictors of mortality (stage 1: OR 2.92 [1.66–5.59], *p* < 0.001; stage 2: OR 2.74 [1.35–5.8], *p* = 0.007; stage 3: OR 4.16 [1.19–14.1], *p* = 0.02). The incidence and severity of procedure-specific AKI showed significant differences between the groups (*p* < 0.001). Conclusions: The occurrence of CSA-AKI is associated with a significantly increased risk of in-hospital mortality, irrespective of stage. Procedure-specific AKI rates differ significantly between types of surgeries. Therefore, consideration of procedure-related risks, as well as early detection, is crucial to improve outcomes after cardiac surgery.

## 1. Introduction

Cardiac surgery-associated acute kidney injury (CSA-AKI) is well known to be associated with increased risks of morbidity, prolonged intensive care unit (ICU)- and hospital-related stays, secondary complications and mortality in the short and long term [1,2,3]. Moreover, it promotes the development of chronic kidney disease and long-term dialysis dependence, generating considerable healthcare costs and resource consumption [4,5,6]. However, despite its disastrous prognostic and economic consequences, awareness of CSA-AKI is still very low [7]. The reported incidence rates vary substantially between 4% and 40%, mostly related to a high degree of underrecognition and the application of different nonstandardized, incomplete definitions, which are mostly dependent on the availability of data [8,9,10]. When the full KDIGO criteria, including urine output, are applied, the incidence of CSA-AKI can reach 60–70% [11,12].

However, detailed knowledge regarding the incidence and impact of CSA-AKI, particularly with respect to different cardiac surgical procedures, is sparse, as obtaining sufficient and robust data remains challenging, especially with respect to the included urine output. With the broad establishment of electronic health records (EHRs), large amounts of digital data are generated. However, data from different clinical EHR systems are often difficult to combine because of interoperability issues [13]. To overcome these obstacles, programming interfaces, decoding and data fusion are crucial for retrieving and assessing these data [14].

This study sought to characterize the incidence and impact of acute kidney injury on mortality depending on different cardiac surgical procedures. Therefore, we applied a dedicated data fusion technique to assess and analyze our large electronic database. To the best of our knowledge, this is the first study in which such large-scale and differentiated AKI and outcome data on cardiac surgical patients have been made available.

## 2. Materials and Methods

### 2.1. Study Design

In this retrospective observational study, we sought to characterize the association between CSA-AKI and the risk of mortality. We hypothesized that 1. the development of CSA-AKI has an impact on mortality and 2. there would be differences regarding severity of disease, i.e., AKI stages, and between types of cardiac surgical procedures. The primary endpoint was CSA-AKI-associated mortality. Secondary endpoints included risk factor analysis for the development of CSA-AKI and procedure-specific AKI incidences and their impact on survival.

### 2.2. Patients

A total of 8564 patients who underwent cardiac surgery at our center between 2017 and 2024 were included in this analysis. Surgical procedures covered the whole spectrum of adult cardiac surgeries except transplantation and were grouped according to the most common and frequent operations: coronary artery bypass grafting with and without the use of cardiopulmonary bypass (OPCAB), aortic and mitral valvular repair and replacement surgeries, tricuspid valve surgeries, aortic surgeries, and combined (valve and/or bypass) procedures. There is a special focus on minimally invasive techniques in our center, i.e., off-pump coronary surgery and the mini-sternotomy or mini-thoracotomy approaches where applicable. Owing to their different natures and impacts on AKI, vascular and wound surgeries were excluded.

### 2.3. Data

**Figure 1:**
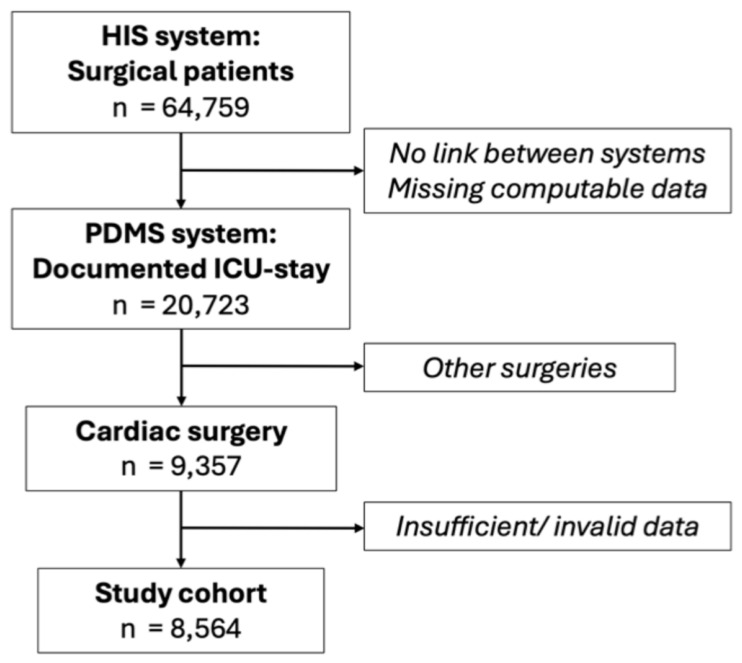
Patient cohort. HIS hospital information system; PDMS patient data management system; ICU intensive care unit; n, number of patients; italics indicate excluded patients.

Data were retrospectively collected from the beginning of the electronic health records at our center in 2017. Algorithm-based techniques were used in two instances. First, for the data retrieval and the data fusion technique used to construct the present dataset, which is based on information originating from two different electronic health record sources. Owing to the lack of interoperability between the primary hospital information system (HIS; iMedOne^®^, Telekom Healthcare Solutions, Bonn, Germany), where surgical procedures outcomes, complications, and length of stay are documented, and the intensive and intermediate care patient data management system (PDMS; Metavision^®^, iMDSoft, Tel Aviv, Israel), where all monitoring and AKI-defining data, e.g., vital signs and urine output, are recorded, data fusion was required for this analysis. Integrating data from various clinical information systems poses significant challenges owing to disparities in data quality and operational protocols across systems, especially concerning the management of entities such as cases or clinical processes. To construct the cohort, we initially gathered patient and surgery data from the primary hospital information system. Subsequently, leveraging insights from physicians, we devised a mapping strategy to the ICU patient documentation management system to determine ICU admission and discharge specifically related to the appropriate surgery. The general mapping of patients between the incorporated systems was facilitated by the use of a unique patient identifier across these systems. As clinical parameters are often redundantly collected, we prioritize the collection from processes known for higher data quality before detecting AKI and performing further analysis. Patients with missing, insufficient, or invalid computable data, especially for the detection of AKI (minimum of two urine output data, one serum creatinine, or one documented dialysis), were excluded. In doing so, over 99% of patients had at least one creatinine observation as well as one urine value normalized by body weight. For other clinical parameters that were less frequently observed, we did not apply any data imputation approach to introduce as little potential bias as possible compared to real-world data. The creation of the study patient cohort is displayed in Figure 1. Second, data were analyzed regarding acute kidney injury according to the full KDIGO criteria, including urine output and KDIGO staging, via algorithm-based big data processing [15]. For identification, grouping, and characterization, ICD-10 and OPS codes were used (Supplementary Tables S1 and S2).

### 2.4. Statistical Analysis

Categorical variables are reported as absolute numbers with percentages and were compared via Pearson’s chi-square test at a significance level of α = 0.05. For continuous variables, median values with interquartile ranges (IQRs) are reported, and the Wilcoxon rank sum test was used for comparison. Confounding risk factor analysis was performed via univariate and multivariate logistic regression models each for the first and maximum AKI stages. Kaplan-Meier survival estimates were compared via the log-rank test. For the main regression analysis, a simulation-based post hoc power calculation was performed. Assuming a mortality rate of 4.4% and a clinically relevant effect size (OR = 1.30), the study achieved approximately 98.60% power at a significance level of 0.005. All statistical analyses were performed via R v4.2.1 (The R Foundation for Statistical Computing, Vienna, Austria).

### 2.5. Ethics Approval and Consent to Participate

The study was approved by the local Ethics Committee of the Medical Faculty of the University of Tuebingen (File-No. 963/2021BO2). The need for informed consent was waived. Approval was also obtained from the local data privacy protection officer. The manuscript was written according to the Strengthening the Reporting of Observational Studies in Epidemiology reporting checklist.

## 3. Results

### 3.1. Baseline and AKI Characteristics

The patients had a median age of 68 [IQR: 60; 75] years, 75.2% were male, and the median body mass index was 26.9 [24.3; 30.1] kg/m^2^. The overall incidence of CSA-AKI was 70.5% (n = 6034 patients). The median duration of AKI development was 18 [11; 27] hours post-operatively until the first AKI stage. The distribution of AKI stages upon first detection was as follows: 88.1% had stage 1 AKI, 10.6% had stage 2 AKI, and 1.4% had stage 3 AKI. The maximum AKI stage was reached after a median of 27 [18; 40] hours post-operatively. Among these patients, 28.1% peaked at AKI stage 1, 54.0% at stage 2, and 17.9% at stage 3. Urine output was the major criterion for AKI detection and accounted for 86.8% of the overall AKI detection. The creatinine- and dialysis-based detection rates were 12.1% and 1.1%, respectively.

Patients who developed CSA-AKI were significantly older (median 70 [61; 76] versus 65 [58; 72] years, *p* < 0.001), heavier (median BMI 27.5 [24.7; 30.9] versus 25.7 [23.4; 28.2] kg/m^2^, *p* < 0.001), and had more comorbidities (median 5 [4; 6] versus 4 [3; 5], *p* < 0.001), which was also reflected by a higher EuroSCORE II (median 3.1 [1.6; 7.3] versus 1.5 [0.9; 2.8], *p* < 0.001). Details about comorbidities can be found in Supplementary Table S3. Overall, these patients had a longer duration of surgery (3.33 [2.8; 4.1] versus 3.03 [2.6; 3.6] hours, *p* < 0.001). In addition, there were longer cardiopulmonary bypass times (122 [87; 164] versus 113 [80; 149] minutes, *p* < 0.001), longer aortic cross-clamp times (78 [56; 104] versus 72 [51; 96] minutes, *p* < 0.001), and longer circulatory arrest times (19 [0; 34] versus 10 [0; 20] minutes, *p* < 0.001). Postoperatively, CSA-AKI patients had significantly prolonged ICU stays (median 3.0 [1.9; 6.1] versus 1.0 [0.9; 1.9] days), hospital lengths of stay (median 9.1 [7.0; 14.1] versus 6.9 [6.0; 8.0]), and a sixfold greater risk of mortality (5.9% versus 0.9%), all *p* < 0.001 (Table 1).

A detailed analysis of risk factors associated with the occurrence of CSA-AKI is presented in the Supplementary Materials (Tables S4 and S5). In brief, several established perioperative variables, including age, comorbidity burden, operative time, and cross-clamp time, were significantly associated with the development of CSA-AKI in uni- and multivariate models.

### 3.2. AKI-Associated Mortality

**Figure 2:**
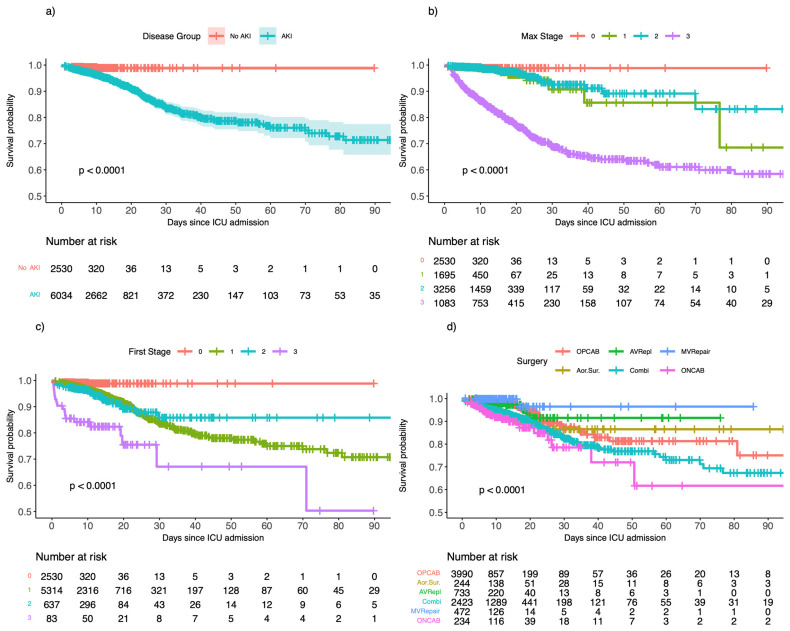
Kaplan-Meier survival estimates for (**a**) AKI patients versus non-AKI patients, (**b**) maximum AKI stages versus 0 non-AKI patients, (**c**) first AKI stages versus 0 non-AKI patients, and (**d**) the six most common cardiac surgeries. Abbreviations: AKI, acute kidney injury; ICU, intensive care unit; Max, maximum; OPCAB, off-pump coronary artery bypass grafting; Aor.Sur., aortic surgery; AVRepl, aortic valve replacement; Combi, combined procedure; MVRepair, mitral valve repair; ONCAB, on-pump coronary artery bypass surgery.

The development of AKI was associated with an increased risk of in-hospital mortality (5.9% versus 0.9%, *p* < 0.0001) (Figure 2a). The mortality rates were 4.4% (375/8564) overall, 0.9% (22/2530) without AKI, 1.5% (26/1695) with stage 1 AKI, 1.8% (59/3256) with stage 2 AKI, and 24.7% (268/1083) with stage 3 AKI. All stages of AKI were associated with an impaired probability of survival, regardless of whether the first or maximum AKI stage was considered (*p* < 0.0001) (Figure 2b,c). Moreover, there were highly significant procedure-specific differences in survival, with the highest survival rates after mitral valve repair and the lowest rates after on-pump coronary artery bypass grafting. Figure 2d shows plotted survival curves for the six most common cardiac surgeries in our cohort, which were, in detail, off- and on-pump coronary artery bypass grafting (OPCAB, ONCAB), combined valvular and bypass procedures (Combi), aortic valve replacements (AVRepl), mitral valve repairs (MVRepair), and aortic surgeries (Aor.Sur.).

### 3.3. Risk Factor Analysis for AKI Mortality

According to the logistic regression risk factor analysis, even after adjustment for potential confounders, CSA-AKI was independently associated with elevated odds of mortality. Notably, when accounting for the first detection of AKI, all stages were predictive of mortality (stage 1: OR 2.92 [1.66; 5.59], *p* < 0.001; stage 2: OR 2.74 [1.34; 5.8], *p* = 0.01; stage 3: OR 4.16 [1.18; 14.01], *p* = 0.02), with first stage AKI 3 achieving the second-highest OR after endocarditis (OR 4.74 [3.17; 7.03]). However, in terms of the maximum AKI stage, only stage 3 remained predictive of mortality (OR 13.14 [7.28; 25.48], *p* < 0.001). The complete results of the risk factor analysis are summarized in Table 2 and in Supplementary Table S4 (first AKI) and Table S5 (max AKI).

### 3.4. Procedure-Specific Mortality

**Figure 3:**
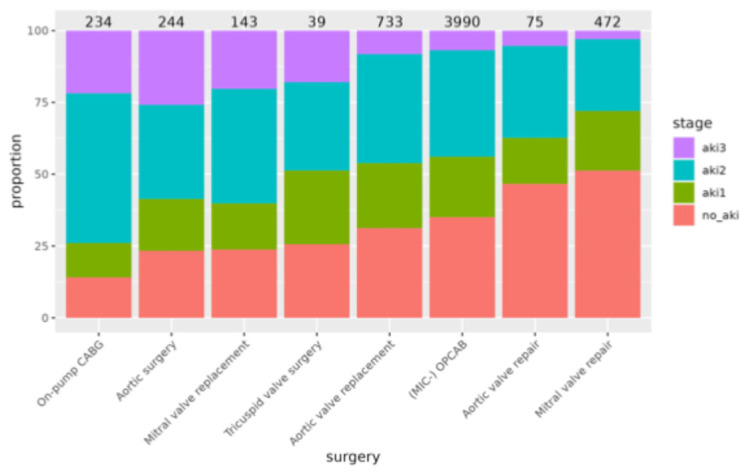
Bar plot displaying the incidence and distribution of AKI stages according to the most common cardiac surgical procedures. Abbreviations: CABG, coronary artery bypass grafting; (MIC) OPCAB, (minimally invasive cardiac surgery) off-pump coronary artery bypass surgery.

The differentiated analysis of specific, most common cardiac operative procedures revealed significant differences in overall AKI rates and AKI stage distributions between types of surgeries (see the bar plot in Figure 3). Combined procedures, on-pump CABG, mitral valve replacement and aortic surgeries had significantly higher-than-average AKI rates and significantly more patients with more severe AKI stages (*p* < 0.001); complete data are provided in Supplementary Table S6. AKI rates were highest after on-pump coronary artery bypass grafting (85.9%) and combined surgeries (81.9%). The lowest AKI rates were observed in isolated mitral (48.7%) and aortic (53.3%) valve repairs and in off-pump coronary artery bypass grafting (65.0%). Patients who developed AKI had significantly higher mortality rates, especially after combined surgery (17.9% vs. 3.4%, *p* < 0.0001), OPCAB (2.5% vs. 0.1%, *p* < 0.0001), and aortic surgery (9.6% vs. 0.0%, *p* = 0.01). The difference in mortality rates reached the borderline significance level for isolated aortic (2.6% vs. 0.4%, *p* = 0.05) and mitral valve replacement (10.1% vs. 0.0%, *p* = 0.05) and was not significantly different for on-pump coronary surgery (11.9% vs. 6.1%, *p* = 0.32) and isolated tricuspid valve surgery (10.3% vs. 0.0%, *p* = 0.29) or aortic (2.5% vs. 0.0%, *p* = 0.35) and mitral (0.9% vs. 0.0%, *p* = 0.15) valve repairs; for complete data, see Table 3.

## 4. Discussion

The risks and impact of cardiac surgery-associated acute kidney injury (CSA-AKI) are still highly underestimated. Our results underscore the increased risk of in-hospital mortality for patients who develop acute kidney injury after cardiac surgery in a large dataset. While AKI was independently associated with increased mortality in our analysis, causality cannot be inferred from this observational study. AKI likely reflects both overall disease severity and a potential contributor to adverse outcomes, given its early occurrence after surgery and its known systemic effects. Therefore, AKI should be interpreted as both a marker of illness severity and a clinically relevant target for early intervention. In our cohort, AKI-associated mortality was 6-fold higher than that in patients without AKI, and for patients with stage 3 AKI, the mortality rate was as high as 24.7%.

In addition, few data on AKI incidence and mortality exist for cardiac surgery-specific patient cohorts. Global AKI reports usually summarize intensive care patients, including major surgeries, and include many different patients [5]. Cardiac surgical publications often refer to small numbers and/or one specific type of surgery, e.g., acute aortic dissection surgery [16]. Nadim et al. reported a 50% mortality rate for cardiovascular patients receiving renal replacement therapy, which is double our data but also includes vascular surgical patients [3].

We based our analysis on a large homogeneous cardiosurgical patient cohort and big data processing, including data fusion from different electronic health records, overcoming the hurdles of missing interoperability between systems. In addition, the full KDIGO criteria, including urine output, were applied for the definition and detection of AKI. The widely differing AKI rates and, consequently, outcome data are also heavily attributed to the use of different criteria, e.g., serum creatinine-based AKI definitions without considering urine output. This severely complicates the comparability of published data and dilutes the enormous impact of AKI on outcomes [12].

Moreover, for the first time, we provide a differentiated analysis of the incidence and stage distributions of AKI according to different cardiac surgical procedures and their correlation with outcomes. Our data revealed the lowest rates of AKI after elective aortic and mitral valve repairs as well as OPCAB coronary bypass surgery and the highest rates for combined procedures and on-pump coronary bypass grafting. The type of surgery has been described as a risk factor for CSA-AKI [17,18]. The risk of CSA-AKI correlates with overall perioperative risk according to common cardiac surgical risk calculators such as the EuroSCORE II [19]. Perioperative risk factors for AKI are multifaceted, as are outcomes in general: preoperative fasting, dehydration, intraoperative blood loss, variable hemodynamics, cardiopulmonary bypass, drugs are all likely to influence the risk of CSA-AKI [20]. However, the significant difference in AKI and mortality rates between off- and on-pump CABGs in our cohort must be interpreted with caution. As a dedicated OPCAB center, we follow a consistent OPCAB-first approach; therefore, the differences in the coronary data are biased by selection and more likely reflect differences in risk profiles between elective low-risk and urgent, emergent, and high-risk coronary surgeries, which are more likely to be performed on-pump. To date, evidence of the role of off-pump versus on-pump coronary artery bypass grafting is conflicting [21,22].

Risk factor analysis revealed that all stages of CSA-AKI, even stage 1 AKI, were predictive of the risk of mortality upon first AKI stage detection. These findings emphasize the importance of early detection and the application of full KDIGO criteria, including urine output, as the majority of stage 1 AKI cases are detected by the urine output criterion. However, stage 1 AKI should not be interpreted as uniformly reflecting structural kidney injury, but rather as a heterogeneous entity ranging from transient functional impairment to early injury. In this context, stage 1 AKI may primarily identify patients at increased risk, particularly those prone to progression to more severe stages. Moreover, stage 1 CSA-AKI can no longer be considered benign and should be acknowledged as a serious complication that requires attention and action. However, when risk factor analysis was focused on the maximum AKI stage, only stage 3 AKI was predictive of the risk of mortality. At first glance, these findings might be conflicting; however, in our opinion, they indicate that lower AKI stages may primarily reflect an early risk state, whereas progression to more severe stages likely indicates both worsening systemic illness and a greater contribution of kidney injury to adverse outcomes. Should we, then, focus only on AKI stages 2 and 3? In contrast, our results emphasize the importance of early surveillance and attention to the so-called lower AKI stages, as kidney-protective measures need to be applied before renal damage is irreversible, with all the detrimental consequences. Recognition of stage 1 AKI patients and kidney-protective intervention could prevent deterioration to AKI stages 2 and 3, which accounted for half of all initially detected stage 1 AKI patients in our cohort. Early detection and implementation of a treatment bundle based on KDIGO recommendations have already been proven to reduce the rates of severe AKI stages [23,24]. Algorithm-based detection or the use of modern biomarkers could enable even earlier action [12]. Therefore, early diagnosis may potentially prevent further deterioration of AKI. Irrespective of causality, AKI identifies a high-risk population early after surgery and thus represents an important window for preventive and supportive interventions.

## 5. Conclusions

Acute kidney injury is one of the most common but still one of the most underestimated complications after cardiac surgery, as it significantly increases the risk of mortality as well as the length of intensive care unit (ICU) and hospital stays at all stages. There are significant differences between procedure-specific AKI rates and associated mortality rates. New developments in early detection methods such as algorithms and biomarkers can potentially help optimize the management of these patients.

### Limitations

This study has several limitations. Although our dataset included a range of established perioperative risk factors for CSA-AKI, such as comorbidities, preoperative renal function, and operative parameters (e.g., cardiopulmonary bypass time and cross-clamp time), it did not capture all clinically relevant determinants in a sufficiently granular manner. In particular, detailed intraoperative hemodynamic data, vasopressor or inotrope use, transfusion requirements, fluid balance, and nephrotoxic medication exposure were not consistently available across data sources. Therefore, residual confounding by unmeasured perioperative severity factors cannot be excluded, and the present analysis should be interpreted as an association study rather than a comprehensive mechanistic model of CSA-AKI.

## Figures and Tables

**Table 1:** Patient characteristics: AKI vs. no AKI.

Variable	Total Cohort	AKI	No AKI	*p* Value
	N = 8564	N = 6034	N = 2530	
Demographics				
Age (years)	68 [60; 75]	70 [61; 76]	65 [58; 72]	<0.001
Male Sex	75.2%	74.7%	76.4%	0.09
BMI (kg/m^2^)	26.9 [24.3; 30.1]	27.5 [24.7; 30.9]	25.7 [23.4; 28.2]	<0.001
Comorbidities (n)	4 [3; 6]	5 [4; 6]	4 [3; 5]	<0.001
Pre-op. creatinine (mg/dL)	1.0 [0.8; 1.1]	1.0 [0.8; 1.2]	0.9 [0.8; 1.1]	<0.001
CKD	7.8%	9.9%	2.8%	<0.001
EuroSCORE II (%)	2.4 [1.3; 5.5]	3.1 [1.6; 7.3]	1.5 [0.9; 2.8]	<0.001
Intraoperative data				
Operation time (hours)	3.3 [2.7; 3.9]	3.3 [2.8; 4.1]	3.0 [2.6; 3.6]	<0.001
CPB time (min)	120 [86; 160]	122 [87; 164]	113 [80; 149]	<0.001
Cross-clamp time (min)	76 [55; 101]	78 [56; 104]	72 [51; 96]	<0.001
Circulatory arrest time (min)	17 [0; 31]	19 [0; 34]	10 [0; 20]	<0.001
Postoperative data				
ICU stay (days)	2.1 [1.1; 4.9]	3.0 [1.9; 6.1]	1.0 [0.9; 1.9]	<0.001
Hospital stay (days)	8.0 [6.8; 12.2]	9.1 [7.0; 14.1]	6.9 [6.0; 8.0]	<0.001
In-hospital mortality	4.4%	5.9%	0.9%	<0.001

Demographic, intraoperative, and postoperative patient characteristics comparing the AKI group and the no AKI group. Categorical data are presented as medians [interquartile ranges], and continuous data are presented as percentages. Abbreviations: AKI, acute kidney injury; BMI, body mass index; CKD, chronic kidney disease; EuroSCORE, European system for cardiac operative risk evaluation; CPB, cardiopulmonary bypass; ICU, intensive care unit.

**Table 2:** Multivariate risk factor analysis.

Variable	First AKI		Max AKI	
	OR [95% CI]	*p* Value	OR [95% CI]	*p* Value
Demographics				
Age	1.06 [1.04; 1.07]	<0.001	1.05 [1.03; 1.07]	<0.001
Female sex	1.36 [0.99; 1.86]	0.053	1.24 [0.88; 1.72]	0.213
BMI	0.96 [0.93; 1]	0.032	0.95 [0.91; 0.98]	0.003
Preop. creatinine	1.21 [1; 1.43]	0.032	1 [0.83; 1.18]	0.987
Preop. Hb	1 [1; 1]	0.156	1 [1; 1]	0.498
Hypertension	0.48 [0.36; 0.66]	<0.001	0.48 [0.34; 0.67]	<0.001
Diabetes mellitus	1.35 [0.94; 1.91]	0.1	1.13 [0.77; 1.64]	0.533
CHF	2.95 [2.08; 4.28]	<0.001	2.75 [1.89; 4.06]	<0.001
Atrial fibrillation	1.22 [0.9; 1.67]	0.208	0.93 [0.66; 1.3]	0.653
Atherosclerosis	1.59 [1.08; 2.31]	0.016	1.82 [1.21; 2.71]	0.004
cAOD	1.98 [1.16; 3.29]	0.01	1.15 [0.65; 1.98]	0.627
COPD	1.89 [1.1; 3.12]	0.017	1.77 [0.99; 3.06]	0.045
CKD	1.45 [0.94; 2.21]	0.087	1.13 [0.71; 1.77]	0.591
Myocardial infarction	1.77 [1.1; 2.81]	0.016	1.48 [0.89; 2.42]	0.122
Obesity	1.07 [0.51; 2.1]	0.844	1.01 [0.46; 2.07]	0.973
Stroke	0.9 [0.05; 5.62]	0.924	0.7 [0.04; 4.34]	0.744
CHD	0.96 [0.68; 1.36]	0.829	0.96 [0.67; 1.38]	0.822
Endocarditis	4.75 [3.18; 7.04]	<0.001	3.65 [2.36; 5.61]	<0.001
Intraoperative data				
Operative time	1.72 [1.42; 2.02]	<0.001	1.63 [1.36; 1.92]	<0.001
CPB time	1 [1; 1.01]	0.14	1 [1; 1.01]	0.157
Number of procedures	0.95 [0.89; 1]	0.059	0.92 [0.87; 0.98]	0.008
Urgency Mid	0.39 [0.2; 0.76]	0.005	0.34 [0.17; 0.69]	0.003
Urgency Normal	0.25 [0.14; 0.44]	<0.001	0.26 [0.14; 0.48]	<0.001
CPB used	1.47 [0.74; 3.15]	0.291	1.31 [0.64; 2.86]	0.475
Cross-clamp time	0.99 [0.99; 1]	0.024	0.99 [0.99; 1]	0.004
Postoperative data				
AKI stage 1	2.93 [1.66; 5.59]	<0.001	0.7 [0.29; 1.62]	0.407
AKI stage 2	2.74 [1.35; 5.8]	0.007	1.38 [0.73; 2.73]	0.337
AKI stage 3	4.16 [1.19; 14.1]	0.023	13.15 [7.29; 25.49]	<0.001

Multivariate risk factor analysis by log. regression model, first and max AKI stages, for mortality after cardiac surgery. Abbreviations: AKI, acute kidney injury; BMI, body mass index; Hb, hemoglobin; cAOD, cerebral arterial occlusive disease; CHF, congestive heart failure; CI, confidence interval; CKD, chronic kidney disease; CPB, cardiopulmonary bypass; CHD, coronary heart disease; COPD, chronic obstructive pulmonary disease; Max. maximum; OR, odds ratio.

**Table 3:** Rates of AKI and mortality for different cardiac surgical procedures.

Cohort	Number	AKI Group		No AKI Group		*p* Value
		AKI Rate	Mortality Rate with AKI	No AKI Rate	Mortality Rate w/o AKI	
All	8564	6034 (70.5%)	353 (5.9%)	2530 (29.5%)	22 (0.9%)	<0.0001
OPCAB	3990	2593 (65.0%)	65 (2.5%)	1397 (35.0%)	2 (0.1%)	<0.0001
ON-CABG	234	201 (85.9%)	24 (11.9%)	33 (14.1%)	2 (6.1%)	0.32
Aortic surgery	244	187 (76.6%)	18 (9.6%)	57 (23.4%)	0 (0.0%)	0.01
AV-Repair	75	40 (53.3%)	1 (2.5%)	35 (46.6%)	0 (0.0%)	0.35
AV-Replacement	733	504 (68.8%)	13 (2.6%)	229 (31.2%)	1 (0.4%)	0.05
MV-Repair	472	230 (48.7%)	2 (0.9%)	242 (51.3%)	0 (0.0%)	0.15
MV-Replacement	143	109 (76.2%)	11 (10.1%)	34 (23.8%)	0 (0.0%)	0.05
TV-Surgery	39	29 (74.4%)	3 (10.3%)	10 (25.6%)	0 (0.0%)	0.29
Combined	2423	1984 (81.9%)	433 (17.9%)	439 (18.1%)	15 (3.4%)	<0.0001

AKI and mortality rates for the overall cohort and the most common cardiac surgical procedures. Abbreviations: AKI, acute kidney injury; OPCAB, off-pump coronary artery bypass surgery; ON-CABG, on-pump coronary artery bypass grafting; AV, aortic valve; MV, mitral valve; TV, tricuspid valve.

## Data Availability

The data presented in this study are available from the corresponding author on reasonable request after internal board review due to ethical and legal constraints.

## Supplementary Materials

The following supporting information can be downloaded at: https://www.mdpi.com/article/10.3390/medsci14020209/s1, Table S1: Comorbidities/ICD-10 Codes; Table S2: Operative procedures/OPS-Codes; List S1: List of confounders for risk factor analysis; Table S3: AKI rates for comorbidities; Table S4: Univariate and multivariate risk analysis considering first AKI stage; Table S5: Univariate and multivariate risk analysis considering max AKI stage; Table S6: AKI rate and stages distribution for common cardiac surgical procedures.

## Abbreviations

The following abbreviations are used in this manuscript:

AKI	Acute Kidney Injury
CABG	Coronary artery bypass grafting
CSA-AKI	Cardiac surgery-associated acute kidney injury
EHR	Electronic health record
EuroSCORE	European system for cardiac operative risk evaluation
HIS	Hospital information system
ICU	Intensive care unit
IQR	Interquartile range
KDIGO	Kidney Disease: Improving Global Outcomes
OPCAB	Off-pump coronary artery bypass grafting
OR	Odds ratio
PDMS	Patient data management system
