# Supplementary material for: Cardiac Surgery-Associated Acute Kidney Injury: Procedure-Specific Incidence and Impact on Mortality—An Algorithm-Based Big Data Fusion Analysis
Source: Med Sci (Basel). 2026 Apr 23;14(2):209. doi: 10.3390/medsci14020209 (PMC13214795; doi:10.3390/medsci14020209)
Supplement: Supplementary file 1 [file medsci-14-00209-s001.zip › medsci-4250728-supplementary.pdf]

Supplement S1

Cardiac Surgery-Associated Acute Kidney Injury: Procedure-Specific Incidence and Impact on Mortality—An Algorithm-Based Big Data Fusion Analysis

Content

- **Table S1.** Comorbidities/ ICD-10 Codes
- **Table S2.** Operative procedures/ OPS-Codes
- **List S1.** List of confounders for risk factor analysis
- **Table S3.** AKI rates for comorbidities
- **Table S4.** Univariate and multivariate risk analysis considering first AKI stage
- **Table S5.** Univariate and multivariate risk analysis considering max AKI stage
- **Table S6.** AKI rate and stages distribution for common cardiac surgical procedures

Table S1. Comorbidities/ ICD-10 Codes

ICD-10 Code	Diagnosis
E10	Type 1 diabetes mellitus
E11	Type 2 diabetes mellitus
E14	Unspecified diabetes mellitus
E66	Obesity
E78	Disorders of lipoprotein and lipidaemias
G47	Sleep disorders
I05	Rheumatic mitral valve diseases
I06	Rheumatic aortic valve diseases
I07	Rheumatic tricuspid valve diseases
I08	Multiple valve diseases
I10	Essential hypertension
I11	Hypertensive heart disease
I12	Hypertensive chronic kidney disease
I13	Hypertensive heart and chronic kidney disease
I15	Secondary hypertension
I21	Acute myocardial infarction
I22	Subsequent ST elevation (STEMI) and non-ST elevation (NSTEMI) myocardial infarction
I23	Certain current complications following ST elevation (STEMI) and non-ST elevation (NSTEMI) myocardial infarction (within the 28 day period)
I25	Chronic ischaemic heart disease
I31	Other diseases of pericardium (Pericarditis constrictiva)
I33	Acute and subacute endocarditis
I34	Nonrheumatic mitral valve disorders
I35	Nonrheumatic aortic valve disorders
I36	Nonrheumatic tricuspid valve disorders
I38	Endocarditis, valve unspecified
I39	Endocarditis and heart valve disorders in diseases classified elsewhere
I42	Cardiomyopathy
I44	Atrioventricular and left bundle-branch block
I46	Cardiac arrest
I48	Atrial fibrillation and flutter
I50	Heart failure
I63	Cerebral infarction
I64	Stroke
I70	Atherosclerosis
I71	Aortic aneurysm and dissection
J44	Other chronic obstructive pulmonary disease
N18	Chronic kidney disease

Table S1. Relevant comorbidities for cardiovascular and renal disease. ICD-10 International Statistical Classification of Diseases and Related Health Problems, 10th version.

Table S2. Operative procedures/ OPS-Codes

OPS	Operative Procedure
5-351.0	Aortic valve replacement
5-351.1	Mitral valve replacement, open/ sternotomy
5-351.2	Mitral valve replacement, minimally-invasive/ thoracoscopy
5-351.4	Trikuspid valve replacement
5-352.0	Redo/ exchange aortic valve prosthesis
5-352.1	Redo/ exchange mitral valve prosthesis
5-352.3	Redo/ exchange tricuspid valve prosthesis
5-353.1	Mitral valve repair, annuloplasty
5-353.2	Mitral valve repair, leaflet repair
5-353.4	Tricuspid valve repair, annuloplasty
5-353.5	Tricuspid valve repair, leaflet repair
5-354.01	Aortic valve: exploration/ thrombectomy
5-354.03	Aortic valve: subvalvular resection (Morrow procedure)
5-354.06	Aortic valve: decalcification
5-354.0a	Aortic valve repair (David procedure)
5-354.11	Mitral valve: exploration/ thrombectomy
5-354.12	Mitral valve: chordae/ papillary muscle repair
5-354.13	Mitral valve: refixation of prosthesis
5-354.14	Mitral valve: decalcification
5-354.32	Tricuspid valve: chordae/ papillary muscle repair
5-356	ASD-closure
5-357.1	Aortic isthmus stenosis repair
5-361	Coronary artery bypass grafting, on-pump
5-362	Minimally invasive coronary artery bypass grafting
5-369.1	Coronary aneurysm - correction
5-369.2	Coronary fistula - closure
5-371.4	Atrial ablation, open/ sternotomy
5-371.5	Atrial ablation, minimally invasive/ thoracoscopy
5-372.4	pericardiectomy, subtotal
5-372.5	pericardiectomy, total
5-374.6	VSD-closure
5-376.4	LVAD implantation
8-851	Cardiopulmonary bypass
8-852.3	ECLS
5-384	Aortic surgery

Table S2. Adult cardiac surgical OPS-codes. OPS Operationen- und Prozeduren-Schluessel (German adaption from the International Classification of Procedures in Medicine (ICPM) of the World Health Organisation (WHO)). ASD atrial septal defect, VSD ventricular septal defect, LVAD left ventricular assist device, ECLS extracorporeal life support.

List S1. List of confounders for risk factor analysis

Age, sex, body mass index, hypertension, diabetes, congestive heart failure, atrial fibrillation, coronary artery disease, atherosclerosis, cerebral arterial occlusive disease, chronic obstructive pulmonary disease, chronic kidney disease, myocardial infarction, apoplex, endocarditis; urgency of surgery, number of procedures, use of cardiopulmonary bypass, extracorporeal life support, operative time, cardiopulmonary bypass time, crossclamp time, weekday of surgery; acute kidney injury, first AKI stage 1, first AKI stage 2, first AKI stage 3, max. AKI stage 1, max. AKI stage 2, max. AKI stage 3.

Table S3. AKI rates for comorbidities

Characteristic	Cohort N=8564	Disease Group AKI N=6034	No AKI N=2530	p-value
CKD	669 (7.8%)	599 (9.9%)	70 (2.8%)	<0.001
CHF	3,996 (46.7%)	3,109 (51.5%)	887 (35.1%)	<0.001
CHD	6,097 (71.2%)	4,320 (71.6%)	1,777 (70.2%)	0.2
Obesity	444 (5.2%)	373 (6.2%)	71 (2.8%)	<0.001
COPD	381 (4.4%)	316 (5.2%)	65 (2.6%)	<0.001
Atherosclerosis	1,132 (13.2%)	844 (14.0%)	288 (11.4%)	0.001
Atrial fibrillation	3,486 (40.7%)	2,775 (46.0%)	711 (28.1%)	<0.001
Stroke	14 (0.2%)	11 (0.2%)	3 (0.1%)	0.8
Myocardial infarction	1,820 (21.3%)	1,409 (23.4%)	411 (16.2%)	<0.001
cAVK	208 (2.4%)	190 (3.1%)	18 (0.7%)	<0.001
Endocarditis	298 (3.5%)	251 (4.2%)	47 (1.9%)	<0.001
Hypertension	6,173 (72.1%)	4,418 (73.2%)	1,755 (69.4%)	<0.001
Diabetes	2,086 (24.4%)	1,542 (25.6%)	544 (21.5%)	<0.001

Table S3. AKI rates for selected comorbidities

Table S4. Univariate and multivariate risk analysis considering first AKI stage

	Univariate		Multivariate	
Variable	OR	p-value	OR	p-value
CHF	6.18 [4.72;8.21]	< 0.001	2.95 [2.08;4.28]	< 0.001
Endocarditis	6.06 [4.42;8.19]	< 0.001	4.75 [3.18;7.04]	< 0.001
Hypertension	0.51 [0.41;0.63]	< 0.001	0.48 [0.36;0.66]	< 0.001
Age	1.05 [1.04;1.06]	< 0.001	1.06 [1.04;1.07]	< 0.001
Urgency Normal	0.16 [0.11;0.22]	< 0.001	0.25 [0.14;0.44]	< 0.001
AKI stage 1	6.75 [4.47;10.74]	< 0.001	2.93 [1.66;5.59]	< 0.001
Operative time	1.76 [1.64;1.89]	< 0.001	1.72 [1.42;2.02]	< 0.001
Urgency Mid	0.31 [0.21;0.46]	< 0.001	0.39 [0.2;0.76]	0.005
AKI stage 2	7.03 [4.15;12.18]	< 0.001	2.74 [1.35;5.8]	0.007
cAOD	6.3 [4.38;8.89]	< 0.001	1.98 [1.16;3.29]	0.01
Atherosclerosis	1.93 [1.5;2.48]	< 0.001	1.59 [1.08;2.31]	0.016
Myocardial infarction	1.8 [1.43;2.24]	< 0.001	1.77 [1.1;2.81]	0.016
COPD	2.17 [1.47;3.11]	< 0.001	1.89 [1.1;3.12]	0.017
AKI stage 3	33.84 [17.34;65.7]	< 0.001	4.16 [1.19;14.1]	0.023
Crossclamp time	1.01 [1.01;1.01]	< 0.001	0.99 [0.99;1]	0.024
BMI	0.97 [0.95;0.99]	0.009	0.96 [0.93;1]	0.032
Pre-op. creatinine	1.4 [1.29;1.51]	< 0.001	1.21 [1;1.43]	0.032
Female sex	1.44 [1.15;1.79]	0.001	1.36 [0.99;1.86]	0.053

Number of procedures	1.22 [1.18;1.26]	< 0.001	0.95 [0.89;1]	0.059
CKD	2.96 [2.25;3.87]	< 0.001	1.45 [0.94;2.21]	0.087
Diabetes mellitus	1.4 [1.11;1.74]	0.004	1.35 [0.94;1.91]	0.1
CPB time	1.01 [1.01;1.01]	< 0.001	1 [1;1.01]	0.14
Pre-op. Hb	1 [1;1.01]	0.038	1 [1;1]	0.156
Atrial fibrillation	2.29 [1.86;2.84]	< 0.001	1.22 [0.9;1.67]	0.208
Pump used	3.39 [2.67;4.35]	< 0.001	1.47 [0.74;3.15]	0.291
CHD	0.92 [0.74;1.16]	0.486	0.96 [0.68;1.36]	0.829
Obesity	0.81 [0.47;1.3]	0.413	1.07 [0.51;2.1]	0.844
Stroke	3.65 [0.57;13.46]	0.091	0.9 [0.05;5.62]	0.924

Table S5. Univariate and multivariate risk analysis considering max AKI stage

	Univariate		Multivariate	
**Variable**	**OR**	**p-value**	**OR**	**p-value**
CHF	6.18 [4.72;8.21]	< 0.001	2.75 [1.89;4.06]	< 0.001
Endocarditis	6.06 [4.42;8.19]	< 0.001	3.65 [2.36;5.61]	< 0.001
Hypertension	0.51 [0.41;0.63]	< 0.001	0.48 [0.34;0.67]	< 0.001
Age	1.05 [1.04;1.06]	< 0.001	1.05 [1.03;1.07]	< 0.001
Urgency Normal	0.16 [0.11;0.22]	< 0.001	0.26 [0.14;0.48]	< 0.001
AKI stage 3	37.49 [24.68;59.98]	< 0.001	13.15 [7.29;25.49]	< 0.001
Operative time	1.76 [1.64;1.89]	< 0.001	1.63 [1.36;1.92]	< 0.001
BMI	0.97 [0.95;0.99]	0.009	0.95 [0.91;0.98]	0.003
Urgency Mid	0.31 [0.21;0.46]	< 0.001	0.34 [0.17;0.69]	0.003
Atherosclerosis	1.93 [1.5;2.48]	< 0.001	1.82 [1.21;2.71]	0.004
Crossclamp time	1.01 [1.01;1.01]	< 0.001	0.99 [0.99;1]	0.004
Number procedures	1.22 [1.18;1.26]	< 0.001	0.92 [0.87;0.98]	0.008
COPD	2.17 [1.47;3.11]	< 0.001	1.77 [0.99;3.06]	0.045
Myocardial infarction	1.8 [1.43;2.24]	< 0.001	1.48 [0.89;2.42]	0.122
CPB time	1.01 [1.01;1.01]	< 0.001	1 [1;1.01]	0.157
Female sex	1.44 [1.15;1.79]	0.001	1.24 [0.88;1.72]	0.213
AKI stage 2	2.1 [1.31;3.51]	0.003	1.38 [0.73;2.73]	0.337
AKI stage 1	1.78 [1;3.17]	0.049	0.7 [0.29;1.62]	0.407

Pump used	3.39 [2.67;4.35]	< 0.001	1.31 [0.64;2.86]	0.475
Pre-op. Hb	1 [1;1.01]	0.038	1 [1;1]	0.498
Diabetes mellitus	1.4 [1.11;1.74]	0.004	1.13 [0.77;1.64]	0.533
CKD	2.96 [2.25;3.87]	< 0.001	1.13 [0.71;1.77]	0.591
cAOD	6.3 [4.38;8.89]	< 0.001	1.15 [0.65;1.98]	0.627
Atrial fibrillation	2.29 [1.86;2.84]	< 0.001	0.93 [0.66;1.3]	0.653
Stroke	3.65 [0.57;13.46]	0.091	0.7 [0.04;4.34]	0.744
CHD	0.92 [0.74;1.16]	0.486	0.96 [0.67;1.38]	0.822
Obesity	0.81 [0.47;1.3]	0.413	1.01 [0.46;2.07]	0.973
Pre-op. creatinine	1.4 [1.29;1.51]	< 0.001	1 [0.83;1.18]	0.987

Table S6. AKI rate and stages distribution for common cardiac surgical procedures

	Cohort	Disease Group			AKI Stages			
Characteristic	N=8564[1]	AKI N=6034[1]	No AKI N=2530[1]	p-value[2]	AKI 1 N=1695[1]	AKI 2 N=3256[1]	AKI 3 N=1083[1]	p-value[3]
Combi	2,423 (28.3%)	1,984 (81.9%)	439 (18.1%)	<0.001	433 (21.8%)	1,027 (51.8%)	524 (26.4%)	<0.001
Mitral valve repair	472 (5.5%)	230 (48.7%)	242 (51.3%)	<0.001	98 (42.6%)	118 (51.3%)	14 (6.1%)	<0.001
(MIC-) OPCAB	3,990 (46.6%)	2,593 (65.0%)	1,397 (35.0%)	<0.001	842 (32.5%)	1,478 (57.0%)	273 (10.5%)	<0.001
On-pump CABG	234 (2.7%)	201 (85.9%)	33 (14.1%)	<0.001	28 (13.9%)	122 (60.7%)	51 (25.4%)	<0.001
Aortic valve repair	75 (0.9%)	40 (53.3%)	35 (46.7%)	0.001	12 (30.0%)	24 (60.0%)	4 (10.0%)	0.4
Mitral valve replacement	70 (0.8%)	60 (85.7%)	10 (14.3%)	0.005	9 (15.0%)	31 (51.7%)	20 (33.3%)	0.003
Aortic surgery	244 (2.8%)	187 (76.6%)	57 (23.4%)	0.032	44 (23.5%)	80 (42.8%)	63 (33.7%)	<0.001
ECLS	17 (0.2%)	15 (88.2%)	2 (11.8%)	0.11	1 (6.7%)	5 (33.3%)	9 (60.0%)	<0.001
HLM	54 (0.6%)	34 (63.0%)	20 (37.0%)	0.2	9 (26.5%)	13 (38.2%)	12 (35.3%)	0.026
Aortic valve replacement	733 (8.6%)	504 (68.8%)	229 (31.2%)	0.3	166 (32.9%)	278 (55.2%)	60 (11.9%)	<0.001
Mitral valve replacement (thora)	73 (0.9%)	49 (67.1%)	24 (32.9%)	0.5	14 (28.6%)	26 (53.1%)	9 (18.4%)	>0.9
Tricuspid valve surgery	39 (0.5%)	29 (74.4%)	10 (25.6%)	0.6	10 (34.5%)	12 (41.4%)	7 (24.1%)	0.4
